# Molecular KRAS ctDNA Predicts Metastases and Survival in Pancreatic Cancer: A Prospective Cohort Study

**DOI:** 10.1245/s10434-025-17036-y

**Published:** 2025-03-11

**Authors:** Jennifer L. Leiting, Roberto Alva-Ruiz, Jennifer A. Yonkus, Amro M. Abdelrahman, Isaac T. Lynch, Danielle M. Carlson, Ryan M. Carr, Diva R. Salomao, Robert R. McWilliams, Patrick P. Starlinger, Cornelius A. Thiels, Travis E. Grotz, Susanne G. Warner, Sean P. Cleary, Michael L. Kendrick, Rory L. Smoot, Benjamin R. Kipp, Mark J. Truty

**Affiliations:** 1 Division of Hepatobiliary and Pancreas Surgery, Department of Surgery, Mayo Clinic, Rochester, MN USA; 2 Department of Medical Oncology, Mayo Clinic, Rochester, MN USA; 3 Department of Laboratory Medicine and Pathology, Mayo Clinic, Rochester, MN USA; 4 Division of General Surgery, University of Toronto, Toronto, ON Canada

**Keywords:** Pancreatic cancer, Pancreatic cancer staging, Molecular staging, Staging laparoscopy, Circulating tumor DNA, KRAS mutation

## Abstract

**Background:**

Patients with pancreatic ductal adenocarcinoma (PDAC) commonly have occult metastatic dissemination and current standard staging methods have significant limitations in identifying these patients. A clinically available assay allows for the identification of mutant KRAS (mKRAS) circulating tumor DNA (ctDNA) from patient plasma and peritoneal fluid that may identify these patients and impact treatment decision making. We investigated the patterns of diagnostic and prognostic capabilities of mKRAS ctDNA in patients with localized PDAC.

**Methods:**

Patients with non-metastatic PDAC were identified and underwent a full staging work-up during their first visit at our institution. Development of metastatic disease and long-term survival outcomes were assessed to compare between the mKRAS testing groups.

**Results:**

Between 2018 and 2022, 785 patients were evaluated. Among the 785 patients who underwent plasma mKRAS testing, 104 were mKRAS positive. Plasma mKRAS-positive patients were more likely to develop metastatic disease and had worse overall survival. In the 419 patients who underwent peritoneal mKRAS, 123 were mKRAS-positive and were more likely to harbor occult metastases or develop peritoneal rather than hematogenous metastases. For patients who underwent both baseline plasma and peritoneal mKRAS testing, any positive mKRAS test regardless of compartment was associated with worse outcomes.

**Conclusions:**

Detection of mKRAS ctDNA in plasma and peritoneal fluid of patients with localized PDAC is not only feasible but also identifies those at high risk of metastatic progression and worse survival outcomes. It allows for better prognostication and can significantly impact subsequent treatment decisions, particularly in patients where an aggressive surgical approach is being considered.

**Supplementary Information:**

The online version contains supplementary material available at 10.1245/s10434-025-17036-y.

Pancreatic ductal adenocarcinoma (PDAC) is a devastating malignancy with poor survival due to its predilection for metastatic dissemination.^1,2^ Accurate staging at diagnosis is critical to guide management decisions, specifically for patients with seemingly localized tumors considered for curative-intent surgical resection. Cross-sectional imaging modalities using computed tomography (CT) and/or magnetic resonance imaging (MRI) are used ubiquitously but fail to identify small-volume disease. Metabolic imaging, such as fluorodeoxyglucose positron emission tomography (FDG-PET), is seldom used in the absence of suspicious radiologic findings on CT or MRI.^3,4^ Elevated carbohydrate antigen (CA) 19-9 levels can correlate with occult metastatic disease, however up to 40% of patients have normal levels or are non-secretors at diagnosis, and non-specific elevations can also be observed in patients with biliary obstruction or inflammation.^5,6^ Staging laparoscopy can identify patients with occult metastases but is only selectively utilized. Furthermore, in the absence of obvious gross visible disease, peritoneal fluid cytology alone has limited sensitivity.^7–10^

Ideally, pancreatic cancer staging should include identifiable tumor-specific biomarkers for more accurate prognostication. Circulating tumor DNA (ctDNA) is nuclear material that, while it cannot make a specific diagnosis, can be readily isolated and quantified.^11,12^ The identification of tumor-specific ctDNA is improved by targeting specific cancer-associated mutations. The Kristen rat sarcoma virus (KRAS) oncogene is an optimal marker because KRAS mutations are infrequently observed in non-malignant entities yet detected in nearly 90% of PDAC tumors.^13,14^

At our institution, a clinically available droplet digital polymerase chain reaction (ddPCR) can identify ctDNA KRAS mutations in patient plasma and peritoneal fluid washings obtained at staging laparoscopy.^10,15,16^ In this present study, we investigated the patterns and prognostic capabilities of mutant KRAS (mKRAS) ctDNA detection in both the plasma and peritoneal fluid of patients with radiologically localized PDAC as part of two separate but overlapping prospective cohort studies.

## Methods

This study was approved by the Mayo Clinic Institutional Review Board (IRB) and was considered minimal risk. All patients had research authorization consent at the time of ctDNA testing and subsequent follow-up. Two separate but overlapping prospective cohort studies were developed using these clinically available ctDNA mKRAS assays. Eligibility included all patients with non-metastatic biopsy-proven PDAC for surgical opinion with subsequent institutional full staging work-up. All patients underwent ctDNA plasma mKRAS and/or peritoneal fluid mKRAS testing at initial presentation to our center. We included both treatment-naïve patients and those who received initial systemic chemotherapy prior to referral. In the absence of metastatic disease, patients underwent neoadjuvant chemotherapy if indicated and were prospectively followed.^17^ Some patients had repeat ctDNA mKRAS testing and these results are also reported.

The ddPCR liquid biopsy isolates ctDNA from patient plasma or peritoneal fluid obtained at staging laparoscopy, and the methodology and validation for the assay have previously been described.^10,15,16^ For plasma specimens, 10 cc of blood was collected and spun down to obtain platelet-poor plasma for ctDNA extraction. For peritoneal specimens, fluid samples from the peritoneal washings were obtained during staging laparoscopy at a separate time point from the planned resection.^7^ During laparoscopy, all peritoneal surfaces were examined for evidence of metastatic disease, with biopsy of any suspicious lesions. The abdomen was instilled with 1 L of normal saline, agitated, and then aspirated and sent for standard cytologic examination and fluid CA19-9 and carcinoembryonic antigen (CEA) level evaluation. For mKRAS ddPCR analysis, two samples of peritoneal fluid were spun down and DNA was extracted according to the manufacturer’s protocol, as previously described.^10^ Patients with either gross disease at laparoscopy or positive cytology were considered to have a positive laparoscopy indicative of metastatic disease.

KRAS test results were categorized as positive or negative based on mKRAS copies/mL detected in either the plasma or peritoneal fluid per test assay standards. Quantitative mutation values (copies/mL) were recorded for the positive specimens. Correlative laboratory values, imaging, and other staging evaluations were recorded within 4 weeks of the mKRAS test and throughout patient follow-up longitudinally. A patient was defined as having metastatic disease if they had documented biopsy-proven disease at laparoscopy (gross metastases or positive peritoneal cytology), on restaging, or obvious radiographic metastatic disease on subsequent follow-up imaging. Any non-peritoneal metastases were classified as hematogenous metastases.

### Statistical Analysis

Continuous variables are presented as mean and standard deviation (SD) if normally distributed, otherwise they are presented as median and interquartile range (IQR). The two-tailed Student’s t-test or Wilcoxon rank-sum test were used for statistical comparison. Welch’s t-test was used for data with unequal population variances. Categorical variables are presented as number and percentage. Fisher’s exact or Pearson’s Chi-square tests were used for statistical comparison. The Kaplan–Meier method was used for univariate survival analysis between the cohorts. Patients were censored if they were alive at the time of their last follow-up. Cox proportional hazard regression was used for multivariable analysis. All analyses were performed using BlueSky Statistics software v. 7.10 (BlueSky Statistics LLC, Chicago, IL, USA). A *p*-value of <0.05 was considered significant.

## Results

From January 2018 to May 2022, 785 patients underwent baseline mKRAS testing assessing plasma and/or peritoneal fluid ctDNA. Of these, 743 (95%) underwent plasma mKRAS testing, 419 (53%) underwent peritoneal mKRAS testing, and 377 (48%) underwent both plasma and peritoneal mKRAS testing. Patients in all three mKRAS testing cohorts were then followed longitudinally over the course of the study. Median follow-up was 16 months for the entire cohort.

### Plasma Mutant KRAS (mKRAS) Cohort

Of the 743 patients who underwent baseline plasma ctDNA mKRAS testing, 639 (86%) were mKRAS-negative and 104 (14%) were mKRAS-positive. Demographics and characteristics of the two groups are seen in Table 1. The majority of patients in both cohorts were borderline resectable or locally advanced. Patients who were mKRAS-negative were more likely to have received neoadjuvant chemotherapy prior to testing compared with those who were treatment-naïve (47% vs. 16%; *p *< 0.001), while plasma mKRAS-positive patients were more likely to have a clinically positive laparoscopy (33% vs. 21%; *p *= 0.032). Overall, 227 (31%) patients underwent subsequent resection with significantly fewer mKRAS-positive patients undergoing resection (11% vs. 34%; *p *< 0.001). Of the resected patients, there was no difference in pathologic outcomes, including treatment response, margins, positive lymph nodes, lymphovascular invasion (LVI), or perineural invasion (PNI) between the two groups (Table 2). However, early postoperative recurrence or death within 6 months of resection was significantly more likely in plasma mKRAS-positive patients compared with mKRAS-negative patients (46% vs. 13%; *p *= 0.013). Overall, plasma mKRAS positivity correlated with subsequent metastatic disease (78% vs. 49%; *p *< 0.001) and more often hematogenous rather than peritoneal metastases (78% vs. 62%; *p *= 0.009). Plasma mKRAS-positive patients were also significantly less likely to be alive on follow-up (20% vs. 58%; *p *< 0.001). The proportion of patients who had evidence of metastatic disease or death was significantly higher in patients who were mKRAS-positive (93% vs. 62%; *p *< 0.001).

**Table 1:** Demographics and characteristics of all plasma and peritoneal mKRAS study patients

	Plasma mKRAS-negative [*n* = 639]	Plasma mKRAS-positive [*n* = 104]	*p*-Value	Peritoneal mKRAS-negative [*n* = 296]	Peritoneal mKRAS-positive [*n* = 123]	*p*-Value
Age, years			0.666			0.285
Median	65	65		64	66	
Range	37–86	40–85		38–86	37–89	
Biological sex			0.831			0.237
Female	280 (44)	44 (42)		143 (48)	51 (42)	
Male	359 (56)	60 (58)		153 (52)	72 (58)	
Race			1.000			0.103
Caucasian	594 (93)	97 (93)		283 (96)	112 (91)	
Other	45 (7)	7 (7)		13 (4)	11 (9)	
Chemotherapy prior to mKRAS testing			< 0.001			0.068
No	340 (53)	87 (84)		146 (49)	73 (59)	
Yes	299 (47)	17 (16)		150 (51)	50 (41)	
Chemotherapy cycles prior to mKRAS testing			< 0.001			0.154
Mean (median)	3.2 (0)	1.3 (0)		3.0 (0)	3.5 (1)	
Range	1–33	1–18		1–20	1–23	
Total number of chemotherapy cycles			0.151			0.005
Mean (median)	9.2 (9)	8.4 (8)		9.6 (10)	8.3 (8)	
Range	0–36	0–35		0–36	0–27	
Anatomic resectability			0.596			0.801
Resectable	62 (10)	12 (12)		13 (4)	6 (5)	
BR/LA	577 (90)	92 (88)		283 (96)	117 (95)	
Serum CA19-9 at diagnosis			0.060			0.870
Non-secretor	44 (7)	7 (6)		21 (7)	10 (8)	
Normal	120 (19)	10 (10)		42 (14)	18 (15)	
Elevated	475 (74)	87 (84)		233 (79)	95 (77)	
Mean (median)	1192 (157)	3852 (425)	< 0.001	1009 (167)	1973 (224)	0.246
Range	2.5–58,370	6–86,000		4–58,370	2.5–44,880	
Serum CA19-9 at mKRAS testing			0.003			0.783
Non-secretor	44 (7)	7 (6)		10 (8)	21 (7)	
Normal	174 (27)	13 (13)		28 (23)	76 (26)	
Elevated	421 (66)	84 (81)		85 (69)	199 (67)	
Mean (median)	936 (83)	2947 (254)	< 0.001	817 (88)	2247 (125)	0.151
Range	3–86,050	6–38,670		4–86,050	3–44,880	
Serum CEA status			0.003			0.031
Normal	335 (52)	38 (37)		168 (57)	55 (45)	
Elevated	304 (48)	66 (63)		128 (43)	68 (55)	
Mean (median)	6.6 (3)	14.9 (4.6)	< 0.001	5.8 (2.7)	13.7 (3.7)	0.009
Range	< 0.1–305	0.3–263		< 0.1–104	0.6–305	
Plasma mKRAS test			< 0.001			0.270
No	0 (0)	0 (0)		26 (9)	23 (19)	
Yes	639 (100)	104 (100)		270 (91)	100 (81)	
Negative	639 (100)	0 (0)		244 (90)	92 (86)	
Positive	0 (0)	104 (100)		26 (10)	15 (14)	
Mean (median) copies	–	515.2 (20.4)		3 (0)	12 (0)	0.204
Range	–	4–43,185		0–108	0–614	
Staging laparoscopy			< 0.001			< 0.001
No	167 (26)	44 (42)		0 (0)	0 (0)	
Yes	472 (74)	60 (58)		296 (100)	123 (100)	
Laparoscopy findings			0.032			< 0.001
Negative	374 (79)	40 (67)		259 (88)	58 (47)	
Positive	98 (21)	20 (33)		37 (12)	65 (53)	
Peritoneal CA19-9 status	[*n* = 303]	[*n* = 37]	0.473			0.004
Normal	192 (63)	21 (57)		217 (73)	72 (59)	
Elevated	111 (37)	16 (42)		79 (27)	51 (41)	
Mean (median)	1762 (0)	133 (0)		294 (0)	4804 (0)	< 0.001
Range	0–507,500	0–1718		0–35,123	0–507,500	
Peritoneal CEA status	[*n* = 303]	[*n* = 37]	0.178			< 0.001
Normal	250 (83)	27 (73)		263 (89)	91 (74)	
Elevated	53 (17)	10 (27)		33 (11)	32 (26)	
Mean (median)	93 (0)	3.24 (0)		0.73 (0)	127 (0)	< 0.001
Range	0–35,123	0–58		0–56	0–25,560	
Peritoneal mKRAS test			0.270			< 0.001
No	303 (47)	63 (61)		0 (0)	0 (0)	
Yes	336 (53)	41 (39)		296 (100)	123 (100)	
Negative	244 (73)	26 (63)		296 (100)^a^	0 (0)	
Positive	92 (27)	15 (37)		0 (0)	123 (100)^b^	
Mean (median) copies	67.3 (3.0)	75.4 (4.7)		–	209.4 (19.75)	
Range	0–12,068	0–1441		–	8.27–12,068	
Resected			< 0.001			0.043
No	423 (66)	93 (89)		203 (69)	97 (79)	
Yes	216 (34)	11 (11)		93 (31)	26 (21)	
Early (< 6 months) postoperative recurrence or death	29 (13)	5 (46)	0.013	13 (14)	5 (19)	0.540
Vital status at F/U			< 0.001			0.046
Dead	269 (42)	83 (80)		99 (33)	54 (44)	
Alive	370 (58)	21 (20)		197 (67)	69 (56)	
Metastatic disease at F/U			< 0.001			< 0.001
No metastatic disease	324 (51)	23 (22)		173 (58)	40 (33)	
Metastatic disease	315 (49)	81 (78)		123 (42)	83 (67)	
Location of metastases			0.009			0.009
Hematogenous	196 (62)	63 (78)		91 (74)	35 (42)	
Peritoneal	119 (38)	18(22)		32 (26)	48 (58)	
Metastases or death at F/U			< 0.001			< 0.001
No	241 (38)	7 (7)		139 (47)	30 (24)	
Yes	398 (62)	97 (93)		157 (53)	93 (76)	

Data are expressed as *n* (%) unless otherwise specified

^a^37 of these 296 patients (13%) had a clinically positive diagnostic laparoscopy

^b^58 of these 123 patients (47%) had a clinically negative diagnostic laparoscopy

*mKRAS* mutant KRAS, *BR* borderline resectable, *LA* locally advanced, *CA19-9* carbohydrate antigen 19-9, *CEA* carcinoembryonic antigen, *F/U* follow-up

**Table 2:** Pathologic outcomes of all plasma and peritoneal mKRAS study patients who underwent resection

	Plasma mKRAS-negative [*n* = 216]	Plasma mKRAS-positive [*n* = 11]	*p*-Value	Peritoneal mKRAS-negative [*n* = 93]	Peritoneal mKRAS-positive [*n* = 26]	*p*-Value
Pathologic treatment effect			0.861			0.045
0—Complete	25 (12)	1 (9)		8 (9)	4 (15)	
1—Major response	48 (22)	4 (36)		23 (25)	3 (11)	
2—Partial response	119 (55)	6 (55)		57 (61)	15 (58)	
3—No response	13 (6)	0 (0)		2 (2)	4 (15)	
No prior therapy	11 (5)	0 (0)		3 (3)	0 (0)	
Positive lymph nodes			0.734			1.000
No	160 (74)	9 (82)		69 (74)	20 (77)	
Yes	56 (26)	2 (18)		24 (26)	6 (23)	
Margins			1.000			0.584
Negative	204 (94)	9 (82)		88 (95)	26 (100)	
Positive	12 (6)	2 (18)		5 (5)	0 (0)	
LVI			0.368			0.357
Negative	188 (87)	11 (100)		78 (84)	24 (92)	
Positive	28 (13)	0 (0)		15 (16)	2 (7)	
PNI			1.000			0.650
Negative	134 (62)	7 (64)		59 (63)	15 (58)	
Positive	82 (38)	4 (36)		34 (37)	11 (42)	

Data are expressed as *n* (%)

*mKRAS* mutant KRAS, *LVI* lymphovascular invasion, *PNI* perineural invasion

**Fig. 1:**
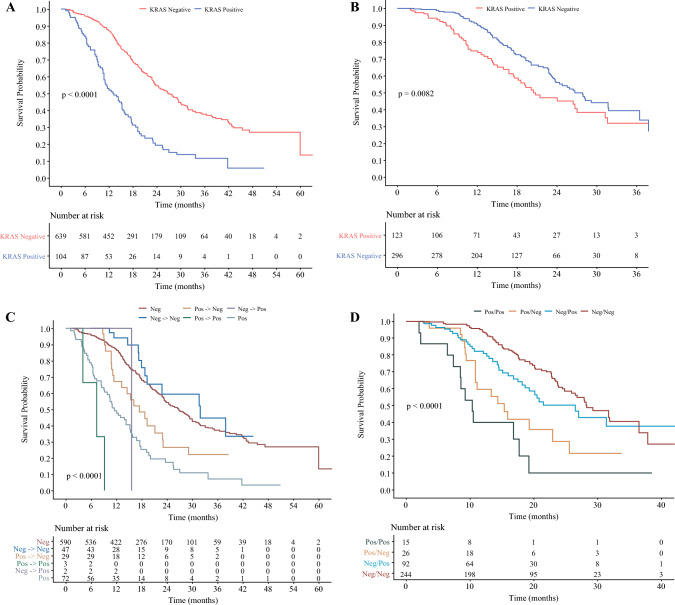
Unadjusted Kaplan-Meier overall survival for patients who underwent plasma KRAS testing (**A**), patients who underwent peritoneal KRAS testing (**B**), patients who underwent multiple plasma KRAS tests (**C**), and patients who underwent both plasma and peritoneal KRAS testing (**D**)

Univariate survival analysis revealed significantly worse OS in patients who were plasma mKRAS-positive compared with patients who were plasma mKRAS-negative (Fig. 1a). Median OS for patients who were plasma mKRAS-positive was just 13.3 months compared with 26.9 months for patients who were plasma mKRAS-negative (*p *< 0.001). Additionally, positive plasma mKRAS status was a significant independent predictor for worse OS (hazard ratio [HR] 2.24; *p *< 0.001) on multivariable analysis (Table 3a).

**Table 3:** Multivariate analysis for overall survival of plasma and peritoneal mKRAS patients

	HR	95% CI	*p*-Value
(a)* Plasma mKRAS patients*			
Age ≥65 years	0.96	0.77–1.19	0.71
Elevated CA19-9	1.37	1.05–1.79	0.021
Positive plasma mKRAS ctDNA	2.24	1.72–2.89	< 0.001
<3 months of chemotherapy	2.97	2.35–3.76	< 0.001
No resection	4.21	3.08–5.73	< 0.001
(b)* Peritoneal mKRAS patients*			
Age ≥65 years	0.87	0.62–1.20	0.394
Elevated CA19-9	1.02	0.67–1.54	0.112
Positive peritoneal mKRAS ctDNA	1.46	1.05–2.05	0.025
<3 months of chemotherapy	2.22	1.55–3.17	< 0.001
No resection	3.68	2.40–5.66	< 0.001
(c)* Combined plasma and peritoneal mKRAS patients*			
Age ≥65 years	1.13	0.80–1.59	0.475
Elevated CA19-9	1.04	0.69–1.58	0.215
<3 months of chemotherapy	3.24	2.16–4.83	< 0.001
No resection	4.04	2.52–6.46	< 0.001
mKRAS ctDNA (ref = Neg/Neg)			
Neg/Pos	1.53	1.02–2.30	0.039
Pos/Neg	3.12	1.82–5.34	< 0.001
Pos/Pos	4.02	2.11–7.64	< 0.001

*mKRAS* mutant KRAS, *HR* hazard ratio, *CI* confidence interval, *CA19-9* carbohydrate antigen 19-9, *ctDNA* circulating tumor DNA, *ref* reference, *Neg/Neg* plasma negative/peritoneal negative, *Neg/Pos* plasma negative/peritoneal positive, *Pos/Neg* plasma positive/peritoneal negative, *Pos/Pos* plasma positive/peritoneal positive

**Fig. 2:**
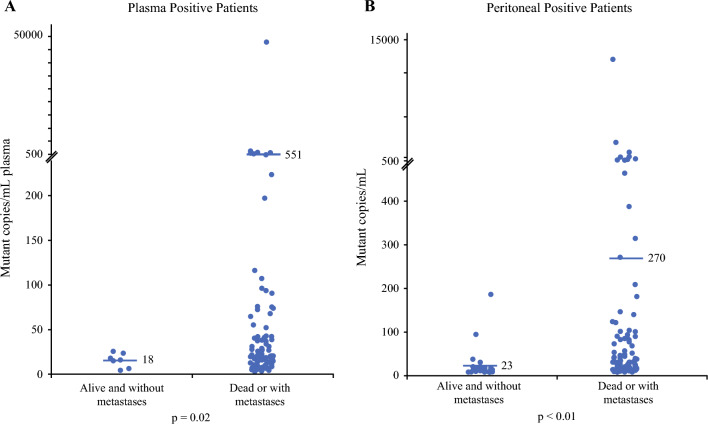
Quantitative plasma (**A**) and peritoneal (**B**) mKRAS results with median values noted by the horizontal line

A small subgroup of patients underwent repeat plasma mKRAS testing (*n *= 82, 11%). Of these patients, 49 were initially mKRAS-negative and 47 (96%) of those patients remained negative on repeat testing after neoadjuvant chemotherapy. Thirty-two patients were initially positive and 29 (91%) of those patients turned negative on repeat testing after receiving neoadjuvant chemotherapy. All patients who were initially plasma mKRAS-positive and underwent resection turned mKRAS-negative with neoadjuvant chemotherapy. On survival analysis, patients who remained positive on repeat testing had a median survival of just 7 months (Fig. 1c). Patients who were initially negative but turned positive had similar overall survival (OS) to patients who were initially positive but turned negative (15.7 months vs. 17.5 months; *p *= 0.98), suggesting a positive plasma mKRAS at any point may be a poor prognostic factor (Table 4). Patients who remained negative on repeat testing had the best OS at 32 months. Comparisons of all plasma testing groups and survival are shown in electronic supplementary material (ESM) Table 1. In plasma mKRAS-positive patients, the quantitative plasma mKRAS copy numbers correlated with metastatic disease development and survival, with higher levels associated with death or metastatic disease (Fig. 2a).

**Table 4:** Comparison of overall survival between plasma mKRAS test groups

Plasma mKRAS test groups	Median overall survival (months)	*p*-Value
Positive → Positive (*n* = 3)	7.4	0.06
Positive (*n* = 72)	11.6	
Negative → Positive (*n* = 2)	15.7	0.98
Positive → Negative (*n* = 29)	17.5	
Negative (*n* = 590)	26.8	0.27
Negative → Negative (*n* = 47)	32.0	

*mKRAS* mutant KRAS

### Peritoneal mKRAS Cohort

Overall, 419 patients underwent baseline peritoneal fluid mKRAS testing and 123 (29%) were mKRAS-positive. Demographics and characteristics of the two groups are seen in Table 1. There was no difference in the number of patients who were concurrently plasma mKRAS-positive (*p *= 0.270). Peritoneal mKRAS-positive patients were much more likely to have clinically positive staging laparoscopy (53% vs. 12%; *p* < 0.001). Overall, 119 (28%) patients underwent subsequent resection, with peritoneal mKRAS-positive patients significantly less likely to undergo resection (21% vs. 31%; *p* = 0.043); however, there was no difference in pathologic outcomes between groups (Table 2). We did not find significant differences in early postoperative recurrence or death within 6 months of resection based on peritoneal mKRAS status (19% vs. 14%; *p* = 0.54) in contrast to resected plasma mKRAS-positive patients. Overall, peritoneal mKRAS positivity correlated with metastatic disease at follow-up (67% vs. 42%; *p* < 0.001) and was more likely peritoneal than hematogenous (58% vs. 26%; *p* = 0.009). Combined metastatic disease or death at follow-up was significantly more likely in those patients with a positive peritoneal mKRAS (76% vs. 53%; *p *< 0.001).

Univariate survival analysis revealed significantly worse OS in patients who were peritoneal mKRAS-positive (Fig. 1b). Median OS was 20.8 months for the peritoneal mKRAS-positive patients compared with 27.9 months for the peritoneal mKRAS-negative patients (*p* = 0.008). On multivariable analysis, peritoneal mKRAS positivity was an independent predictor of worse OS (HR 1.46; *p* = 0.025) (Table 3b). Similar to the plasma mKRAS cohort, quantitative peritoneal mKRAS copy numbers detected correlated with metastases and survival, with higher levels associated with death or metastatic disease (Fig. 2b).

### Combined Plasma and Peritoneal mKRAS Cohort

Overall, 377 patients underwent both baseline plasma and peritoneal mKRAS testing concurrently as they were enrolled in both prospective studies. For analysis, these patients were organized into four groups based on compartment-specific mKRAS status: positive plasma and peritoneal mKRAS (Pos/Pos); positive plasma mKRAS and negative peritoneal mKRAS (Pos/Neg); negative plasma mKRAS and positive peritoneal mKRAS (Neg/Pos); or negative plasma and peritoneal mKRAS (Neg/Neg). The majority of patients were Neg/Neg (*n* = 244, 65%), with just 4% (*n* = 15) being Pos/Pos.

On univariable survival analysis, patients who were Pos/Pos had the worst survival, with a median OS of just 10.4 months (Fig. 1d). Patients who were Pos/Neg had a median OS of 15.4 months and Neg/Pos patients had a median OS of 26.5 months. Patients who were Neg/Neg had the longest median OS of 28.4 months (*p *< 0.001). Any positive mKRAS test regardless of compartment was associated with worse outcomes (Table 5a). Furthermore, the presence and location of metastases varied on the compartment-specific mKRAS status. Patients with a positive plasma mKRAS were more likely to develop hematogenous metastases (56% vs. 27%; *p *< 0.001) and those with a positive peritoneal mKRAS were more likely to develop peritoneal metastases (40% vs. 11%; *p *< 0.001) [Table 5b]. In the Pos/Pos group, 93% of patients developed metastases or had died at last follow-up compared with only 50% in the Neg/Neg group (*p* < 0.001) (Table 5b).

**Table 5:** Combined mKRAS compartment status for (a) overall survival and (b) metastases or death, type of metastases, and mKRAS copy number

	Plasma Pos/Peritoneal Pos [*n* = 15]	Plasma Pos/Peritoneal Neg [*n* = 26]	Plasma Neg/Peritoneal Pos [*n* = 92]	Plasma Neg/Peritoneal Neg [*n* = 244]	*p*-Value
(b) Combined mKRAS compartment status					
Metastases or death (%)	14 (93)	21 (81)	68 (74)	123 (50)	< 0.001
Metastasis type					< 0.001
Hematogenous (%)	8 (53)	15 (58)	22 (24)	68 (28)	
Peritoneal (%)	5 (33)	3 (12)	38 (41)	27 (11)	
No metastases (%)	2 (13)	8 (31)	32 (35)	149 (61)	
Plasma mKRAS copy number	87.3	29.6	–	–	< 0.001
Peritoneal mKRAS copy number	201.6	–	239.9	–	< 0.001

*OS* overall survival, *Pos* Positive, *Neg* Negative

^*^p < 0.01

On multivariable analysis, mKRAS status was a significant predictor of worse OS, with increasing survival hazard based on compartment-specific mKRAS status (Table 3c), with worse outcomes in the Pos/Pos group (HR 4.02; *p *< 0.001).

Finally, we assessed the diagnostic accuracy of both plasma and peritoneal mKRAS compared with plasma and peritoneal tumor markers to predict metastases and death (ESM Table 2). Plasma mKRAS positivity was less sensitive than plasma CA19-9 levels but was significantly more specific. Peritoneal mKRAS positivity had similar low sensitivity but high specificity comparable with peritoneal fluid CA19-9 and CEA levels.

## Discussion

Despite modern staging examinations at diagnosis and increasing utilization of neoadjuvant chemotherapy for borderline and locally advanced PDAC prior to resection, metastatic recurrence is the expectation and this supports the concept that almost all PDAC patients have occult metastatic disease regardless of initial staging. There is a subset of patients who develop rapid metastatic progression despite seemingly localized disease without other associated risk factors.^18^, ^19^ In this current series, the incidence of detectable ctDNA KRAS mutations in the peripheral blood of patients with radiologically localized disease is approximately 15%, which is slightly lower than what has been found in other studies.^16,20^ Interestingly, this is as high as 20% in chemotherapy-naive patients but drops to 5% (*p* < 0.001) in those who received chemotherapy *prior* to mKRAS testing in our series, suggesting the timing of ctDNA assays is essential and has critical implications on any proposed trials looking at plasma ctDNA and the timing of such collected assays.

We found that patients with a positive plasma mKRAS do worse than patients with a negative plasma mKRAS and that these patients have a higher likelihood of hematogenous metastases. Even patients who convert to negative with induction chemotherapy trended towards worse outcomes compared with patients who were baseline negative. Similarly, we found that one-third of patients had detectable peritoneal mKRAS ctDNA at staging laparoscopy, and these patients do worse overall than patients with a negative peritoneal mKRAS with a higher propensity towards peritoneal metastases. Lastly, there seems to be a compounding effect of positive mKRAS with increasing survival hazard depending on the compartment-specific site of mKRAS positivity. Interestingly, there was minimal correlation between plasma and peritoneal mKRAS results. This may be because hematogenous metastases would likely only be identified with a plasma-specific assay and peritoneal dissemination with a peritoneal fluid-specific assay, and this is supported by the current known mechanisms of metastases for each compartment.

Our understanding of how quantitative mKRAS values change over time, and its subsequent clinical implication, continues to evolve. One study found that quantitative changes in mKRAS ctDNA was associated with response to therapy in patients with metastatic PDAC.^21^ In our small subset analysis of patients who received a second plasma mKRAS test within the course of their treatment, we found that a patient’s mKRAS status can change over time. Other studies have found a significant positive correlation between tumor burden and ctDNA levels in patients with PDAC, and significantly worse outcomes in those with detectable ctDNA.^22–25^ In our study, patients who converted from plasma-positive to plasma-negative mKRAS experienced outcomes closer to patients who were baseline plasma positive rather than initially plasma negative. Further study and analysis are needed for this cohort of patients to identify the impact of an initially positive plasma KRAS during a treatment course. Patients with detectable mKRAS in either compartment were far less likely to undergo resection after neoadjuvant therapy due to progressive metastatic disease. Of those who did undergo resection, there was a significantly higher rate of early postoperative recurrence and death in resected patients who were initially plasma mKRAS-positive but turned mKRAS-negative with neoadjuvant chemotherapy. Further investigations are needed to determine how best to counsel these patients given potential worse outcomes after seemingly curative surgery.

The molecular analysis of peritoneal fluid may further assist to identify patients who may not benefit from an extended resection.^26,27^ Our group showed that in an initial pilot of peritoneal fluid ctDNA detection feasibility looking at 136 patients, while only 24% of patients had a clinically positive diagnostic laparoscopy, a higher proportion of patients had detectable mutant mKRAS in their peritoneal fluid.^7^ This current larger series suggests that patients with positive peritoneal but negative plasma mKRAS are more likely to develop peritoneal metastases rather than hematogenous metastases. When compared with patients who were negative for both plasma and peritoneal mKRAS, we found an increasing risk of worse survival in patients who were only positive in the peritoneal fluid, followed by patients who were only positive in the plasma, with worst outcomes being seen in patients who were positive for both. This would suggest that plasma positivity confers a slightly higher risk of worse outcomes than peritoneal positivity alone.

There are several limitations to our study. Although this current study is a prospective cohort with ctDNA drawn at baseline, it is a retrospective analysis of subsequent outcomes after that initial mKRAS test, and is thus associated with significant referral and selection bias. All patients in this study were initially radiologically non-metastatic, hence the results only apply to this subset. Furthermore, the majority of cases in this series had more anatomically advanced tumors (borderline and locally advanced) that are unique to our referral practice, thus results cannot be applied to resectable tumors in general. This study is also limited by its relatively short median follow-up, however despite this, we were still able to show significant associations with survival outcomes, hence the potential prognostic power of a positive mKRAS test. Although other commercially available ctDNA platforms exist, results can only be applied to our institutional assay alone, which is clinically available to all centers as a send-out test. In addition, we did not formally assess primary tumor mKRAS status and compare it with their ctDNA status. However, given the prevalence of KRAS mutations in PDAC, we can assume about 10% will have negative mKRAS ctDNA.

## Conclusions

Molecular-based assays to predict patients at risk of early metastatic progression are critically needed, specifically in patients considered for surgical resection. We have shown that detection of mKRAS ctDNA in plasma and peritoneal fluid can identify patients at risk of metastatic progression and worse survival. As positive mKRAS status correlates with worse outcomes, even in those subsequently undergoing surgical resection, these patients may require extended neoadjuvant chemotherapy and be counseled on the predicted utility or futility of subsequent surgery in the absence of obvious metastatic progression during neoadjuvant therapy. Additional investigation is needed to determine the longitudinal implications of quantitative ctDNA measurements and how to incorporate them into a standard protocol.

## Supplementary Information

Below is the link to the electronic supplementary material.

Supplementary file1 (DOCX 14 KB)
