# Supplementary material for: Molecular KRAS ctDNA Predicts Metastases and Survival in Pancreatic Cancer: A Prospective Cohort Study
Source: Ann Surg Oncol. 2025 Mar 11;32(6):4453–63. doi: 10.1245/s10434-025-17036-y (PMC12049301; doi:10.1245/s10434-025-17036-y)
Supplement: Supplementary file 1 — Supplementary file1 (DOCX 14 KB) [file 10434_2025_17036_MOESM1_ESM.docx]

**Supplemental Table 1.** Median Overall Survival Comparison between Plasma mKRAS Groups

| **Plasma mKRAS Testing Groups** | | **p** |
| --- | --- | --- |
| Positive → Positive | Positive | 0.06 |
| Positive → Positive | Negative → Positive | 0.06 |
| Positive → Positive | Positive → Negative | **<0.01** |
| Positive → Positive | Negative | **<0.01** |
| Positive → Positive | Negative → Negative | **<0.01** |
| Positive | Negative → Positive | 0.53 |
| Positive | Positive → Negative | **0.02** |
| Positive | Negative | **<0.01** |
| Positive | Negative → Negative | **<0.01** |
| Negative → Positive | Positive → Negative | 0.98 |
| Negative → Positive | Negative | 0.51 |
| Negative → Positive | Negative → Negative | 0.07 |
| Positive → Negative | Negative | **0.01** |
| Positive → Negative | Negative → Negative | **0.01** |
| Negative | Negative → Negative | 0.27 |

Abbreviations: mKRAS, mutant KRAS

**Supplemental Table 2.** Diagnostic Testing Parameters for Plasma and Peritoneal mKRAS Compared to Standard Laboratory Tests

| **Variable** | **Metastases or Death** | | | |
| --- | --- | --- | --- | --- |
|  | **Sensitivity** | **Specificity** | **PPV** | **NPV** |
| Plasma KRAS Positive | 20% | 97% | 93% | 38% |
| Elevated Plasma CA 19-9 | 70% | 35% | 68% | 37% |
| Elevated Plasma CEA | 55% | 60% | 73% | 40% |
| Peritoneal KRAS Positive | 37% | 82% | 76% | 47% |
| Elevated Peritoneal CA 19-9 | 39% | 81% | 75% | 47% |
| Elevated Peritoneal CEA | 22% | 95% | 95% | 45% |

Abbreviations: mKRAS, mutant KRAS; PPV, positive predictive value; NPV, negative predictive value
